# Effects of Lifetime Unemployment Experience and Job Insecurity on Two-Year Risk of Physician-Diagnosed Incident Depression in the German Working Population

**DOI:** 10.3390/ijerph14080904

**Published:** 2017-08-11

**Authors:** Natalia Wege, Peter Angerer, Jian Li

**Affiliations:** 1 Institute of Occupational, Social and Environmental Medicine, Centre of Health and Society (CHS), Faculty of Medicine, University of Düsseldorf, 40225 Düsseldorf, Germany; peter.angerer@uni-duesseldorf.de (P.A.); jian.li@uni-duesseldorf.de (J.L.); 2 Department of Psychiatry and Psychotherapy, Faculty of Medicine, University of Düsseldorf, 40225 Düsseldorf, Germany

**Keywords:** job insecurity, unemployment, incident depression, working population

## Abstract

Unemployment and job insecurity have been reported to be associated with a higher risk of depression. The purpose of this study was to evaluate the separate and combined effects of lifetime unemployment experience and job insecurity on the incidence of depression in an unselected working population in Germany. Data from the German Socio-Economic Panel (GSOEP) study were used, as was a final sample of those currently employed, with complete data at baseline (2009) and follow-up (2011) restricted to those free of depression in 2009 (*n* = 7073). Poisson regression analysis was applied to test the prospective associations between unemployment, job insecurity, and a two-year incident of depression. Results showed that the experience of unemployment and perceived job insecurity were significantly associated with a higher risk of depression during the two-year follow-up (risk ratios 1.64; 95% confidence intervals (1.16, 2.31) and risk ratios 1.48; 95% confidence intervals (1.13, 1.92), respectively). Notably, the strongest risk was observed among participants with insecure jobs and past long-term unemployment (risk ratios 2.15; 95% confidence intervals (1.32; 3.52)). In conclusion, even during employment, the experience of lifetime unemployment led to a higher risk of depression. The combination of previous unemployment experience and anticipated job insecurity increased the risk of developing depression. Results support health promotion with special emphasis on unemployment and precarious working conditions.

## 1. Introduction

Participation in paid work on a sustained basis is one of an individual’s core needs. It determines individual economic conditions, social status, socio-economic position, and may influence health through various pathways. A recent systematic meta-review on employment and mental health posed that “…having a job is associated with a greater sense of autonomy, improved self-reported well-being, reduced depression and anxiety symptoms, increased access to resources to cope with demands, enhanced social status and unique opportunities for personal development and mental health promotion” [1].

Despite relatively stable labor market developments in Germany after the economic crisis in 2008, the level of unemployment has remained relatively high, with 1.76 million unemployed in 2017 [2]. The adverse health effect of unemployment has been well documented [3]. Job loss was found to be associated with elevated risks of impaired self-rated health [4], the incidence of depression [5], anxiety [6], and suicide [7]. In a meta-analysis, McKee-Ryan et al. [8] summarized the results of epidemiological studies on unemployment and mental health as follows: (1) unemployed workers revealed significantly lower mental health; (2) a significant reduction in mental health following job displacement was demonstrated; and (3) a significant improvement in mental health was observed when workers became reemployed. A dose-response relationship between the duration of unemployment and psychosocial outcomes was recently reported, with poorer outcomes observed in those with experiences of longer unemployment [9].

The current trend toward alternative forms of employment in Europe, such as temporary contracts or outsourcing, has increased job insecurity among employees [10]. This development was accelerated in most European countries by the economic crisis beginning in 2008 [11]. Job insecurity, defined as a “perceived powerlessness to maintain the desired continuity in a threatened job situation” [12], has been shown as a further risk factor for poor mental health [13], particularly for depression [14] and suicidal ideation [15].

Interestingly, several studies have compared the size of the effects produced separately by unemployment and job insecurity. In a recent systematic review and meta-analysis of 20 prospective observational cohort studies, both job insecurity and unemployment were significantly related to a higher risk of self-reported depressive symptoms or self-reported depression [16]. The authors concluded that by comparing both stressors, “…job insecurity can pose a comparable and even modestly increased risk of subsequent depressive symptoms” [16]. However, research on the combined effects of unemployment and job insecurity is still sparse. To the best of our knowledge, only one study has examined the separate and combined effects of lifetime unemployment experience and job insecurity on the intake of antidepressant medication as an indicator of depression [17]. However, this prospective study from Denmark ran the risk of misclassification due to the fact that many clinical conditions other than depression are also eligible for antidepressant use, and not all individuals with depression are treated with antidepressants. Moreover, the difference in the social security systems in Denmark and Germany might influence the relationship between unemployment and mental health [18].

Therefore, our aim was to test the separate and combined effects of unemployment and job insecurity on the onset of physician-diagnosed depression in a large working population in Germany. More specifically, the following questions were asked: (1) Is there a difference in the risk of depression between working individuals with and without unemployment experience in this population? (2) Does job insecurity at the baseline increase the risk of depression at follow-up? (3) How does the experience of unemployment interplay with job insecurity to impair mental health in terms of depression?

## 2. Materials and Methods

### 2.1. Materials and Study Sample

Data on an unselected population sample from the German Socio-Economic Panel (GSOEP) study were used. In the GSOEP, information has been collected annually since 1984 through face-to-face interviews [19]. Household members aged 18 years and older were eligible for participation. The GSOEP complies with national laws, and informed consent was obtained from all participants [20]. Information on depression has been collected since 2009. A total of 20,792 individuals participated in the 2009 survey, and we restricted this sample to those who were employed (*n* = 11,381). A subsample (*n* = 9464) had complete data on all variables used for the current analyses (see below). Overall, 7354 participants with complete data were followed up in 2011 (response rate = 77.7%). Dropout analyses were also performed. Within the employed individuals (*n* = 11,381), those included in the current study (*n* = 7354) and those excluded due to missing data or non-participation at follow-up (*n* = 4027) differed in some characteristics. Excluded individuals were younger, received less education, had lower incomes, had a lower percentage of permanent contracts, were more likely to be unmarried, and were smokers. However, there were no systematic differences on gender, sport, self-rated health or, most importantly, with regard to years of unemployment experience, job insecurity, or the prevalence of depression. The final sample was further restricted to those free of depression in 2009 (*n* = 7073).

### 2.2. Measures of Unemployment Experience, Job Insecurity, and Depression

Cumulative time of unemployment prior to 2009 was retrieved from the biography and life history data of all GSOEP participants. Two categories were defined: (1) never or short-term (no more than two years); and (2) long-term unemployment with two and more years of unemployment experience. The two-year cutoff point for long-term unemployment experience was defined as critical due to the specific social system in Germany, where, in general, unemployment benefits (Arbeitslosengeld) allow for 67% (60% for those without children) of the insured’s net earnings to be paid for up to 24 months.

Job insecurity was assessed by a question on how participants rated the probability that they would lose their job within the next two years. Respondents answered (in 10% increments) using a scale from 0–100%. Two categories of job insecurity were defined: (1) none or low: <50%; and (2) high: >50%, in line with previous GSOEP research [21].

For further statistical analysis, a combined variable with four categories was constructed: (1) never or short-term unemployment and none or low job insecurity; (2) never or short-term unemployment and high job insecurity; (3) long-term unemployment and none or low job insecurity; and (4) long-term unemployment and high job insecurity.

Depression was included in a tick-off list of conditions introduced by the question, “Has a physician ever diagnosed you with one or several of the following conditions?” The incident of depression was defined as an affirmative reply to this question in 2011.

### 2.3. Confounding Variables

Factors such as age, gender, marital status, and socio-economic position have been found to be related to job insecurity [10], and have also been shown to be associated with depression [5]. Thus, we considered age, gender, marital status, education, income, and contract type as potential confounders (Tables 2 and 3). Of these, age, gender, marital status, and socio-economic variables were used in the adjusted models, as they were associated with both employment conditions and depression (Model I and Model II). We further explored whether lifestyle factors such as smoking, physical exercise, BMI, and alcohol consumption influenced the relationship (Model III), as these health behaviors are associated with precarious working conditions and depression [22,23].

Information on the relevant confounding factors such as age, gender, marital status, education, income, contract type, and self-rated health was collected in 2009. Two alternative indicators of socio-economic position were included in the analysis: level of education and income. Education was classified according to the International Standard Classification of Education as total years of formal education, combining school and vocational training. Income was personal net income per month. Contract type included two categories: (1) permanent contracts and (2) non-permanent contracts. The single-item question on self-rated health has been used as a global measure of general health and a strong and independent predictor of morbidity and mortality [24]. Self-rated health was measured by a standard five-point Likert scale item (“very good”, “good”, “fair”, “poor”, and “very poor”, with less than good indicating poor health). Two categories were constructed for further analyses: (1) good; and (2) poor. Due to the lack of health-related behavior variables in the 2009 surveys, data on smoking (non-smokers/smokers), alcohol consumption (never to occasionally/regular), physical exercise (never/less than once a week/once a week or more), and body mass index (BMI, normal/overweight/obese) in 2008 were used alternatively.

### 2.4. Data Analysis

To describe the sample, descriptive statistics as well as the student’s *t* test (for continuous variables) and χ2 analysis (for categorical variables) were computed, between different levels of unemployment experience and job insecurity. The associations between unemployment experience/job insecurity at the baseline and depression incidence at follow-up was estimated by using a Poisson regression with a log-link function and empirical (robust) variance in SAS and expressed as risk ratios (RRs) with 95% confidence intervals (CIs) [25]. We controlled the analysis first for age and gender at the baseline (Model I); followed by an additional adjustment for marital status, education, income, and contract type at the baseline (Model II); smoking, alcohol consumption, physical exercise, and BMI at the baseline (Model III); and finally for self-rated health at the baseline (Model IV).

### 2.5. Availability of Data

The German Socio-Economic Panel is a public-use dataset, which can be obtained from the Deutsches Institut für Wirtschaftsforschung (DIW), Berlin.

### 2.6. Ethics

All procedures contributing to this work complied with the ethical standards of the relevant national and institutional committees on human experimentation and with the Helsinki Declaration of 1975, as revised in 2008.

## 3. Results

### 3.1. Descriptive Statistics

Study participants were middle-aged; and about half of the sample was female. In total, 244 cases of depression occurred between 2009 and 2011. Table 1 provides the main descriptive characteristics of the study population. In all, 23.2% of participants reported high job insecurity (*n* = 1645), and 9.3% of participants (*n* = 661) reported having long-term unemployment experience. Significant differences in socio-economic characteristics and health-related behaviors were found between groups with low and high job insecurity. Individuals reporting high job insecurity were significantly younger, less educated, had lower net incomes, and more frequently had non-permanent contracts. Moreover, they were more likely to be smokers, reported less sporting activity, and had a poorer self-rated health. Participants with long-term unemployment experience were significantly older, more likely to be women, less educated, had lower net-incomes and higher separating/divorce parentage, and reported less physical activity and poorer self-rated health.

### 3.2. Multivariate Analyses

The separate effects of unemployment experience and job insecurity at the baseline and those of depression at follow-up are displayed in Table 2. The risk of depression during the two-year follow-up was significantly elevated with long-term unemployment experience and with the perception of job insecurity. These health effects were not substantially reduced after adjustment for socio-demographic and socio-economic variables (Model II), nor for health-related behaviors (Model III). However, further adjustment for self-rated health at the baseline slightly attenuated the associations. The risk of depression remained significantly elevated at 64% with long-term unemployment experience, or 48% with the perception of job insecurity in the fully adjusted models.

In Table 3, the combined effects of unemployment experience and job insecurity at the baseline on depression at follow-up are presented. Both variables (independently and significantly) led to a higher risk of depression in the fully adjusted models. The highest risk of depression was observed in working individuals with long-term unemployment experience in combination with insecure jobs. Attenuation of the risk ratio in the fully adjusted model was also observed in cases of combined unemployment experience and job insecurity.

Additionally, stratified analysis by gender was performed, and the pattern was different between men and women. Unemployment experience had a stronger impact on the mental health of men (RR 2.11; 95% CI (1.15–3.88)) in comparison to women (RR 1.40; 95% CI (0.92–2.12)). However, the relationship between job insecurity and depression was stronger among women (RR 1.58; 95% CI (1.14–2.18)) than that among men (RR 1.33 95% CI (0.84–2.13)) in the fully adjusted model (detailed data can be delivered on request).

## 4. Discussion

We found a significantly higher risk of depression after two years of observation in currently working individuals with past long-term unemployment experience. Reported job insecurity was significantly associated with a higher risk of depression after two years. Separate effects of unemployment experience and job insecurity were observed, in line with the previous findings reported in Reference [16]. This current German study indicated, in accordance with the Danish study [17], that the highest risk of depression occurred in the group of working individuals with long-term unemployment experience in combination with insecure jobs. The adverse effects of previous lifetime unemployment still remained, even for those currently in a secure job, but were most obvious among those with insecure working conditions.

Generally, it has been assumed that unemployment may lead to poor mental health due to a lack of resources. According to the transactional model, a negative association of full-time employment with depression has been shown to be fully mediated by social support, less coping with drugs/medication, and less distress [26]. Furthermore, an explanation for the long-term effect of unemployment (even among currently employed persons) could be a “scarring effect”, as identified by the longitudinal National Child Development Study (NCDS) [27]. Those unemployed for longer periods throughout adulthood still experienced raised levels of distress at age 50, demonstrating that unemployment was likely to have long-term societal effects. Similarly, experiences of youth unemployment were clearly shown to be connected with deteriorating mental health at three target ages; 21, 30, and 42 years in a 27-year prospective “Northern Swedish Cohort” (NSC) study [28]. The combined effect of past unemployment experience and future job insecurity could be explained by the concept of “cognitive employment insecurity” [29], as it can be expected that the experience of unemployment will increase an individual’s “employment insecurity”. Supposedly, individuals with insecure jobs (“job insecurity”) as well as a significant period of unemployment in the past may believe that they will lose their job in the near future, and will not be able to find a new one. Previous negative employment experiences may increase the effects of job insecurity on individuals [30,31].

Several limitations should be mentioned when interpreting the results. First, a healthy worker effect needs to be considered, as those with extremely adverse employment experience, precarious working conditions, and/or severe mental disorders might not have been followed up. Thus, the observed associations could be underestimated. Second, though we excluded subjects with doctor-diagnosed depression at the baseline, it is highly possible that some individuals with non-diagnosed depression (i.e., depressive symptoms) were still included in our analysis. The prevalence of depressive symptoms may have been significantly higher in the long-term unemployment and job insecurity groups due to previous experience. It has been suggested that persons with persistent or recurrent depressive symptoms might have an increased risk of negative employment consequence [32], and ill mental health among unemployed individuals was associated with lower levels of reemployment after one year [33]. This methodological issue should also be considered by interpreting current results in comparison with other studies, mostly using different self-reported questionnaires [9,13,16] and clinical interviews [9,14] for depression measurement.

Due to the lack of solid measurements for depressive symptoms, we used a proxy health indicator, self-rated health, in the adjusted analyses (see Model IV). The outcome of our study was not based on systematic medical records; however, a validation study revealed a high rate of confirmed diagnosis among participants with self-reported physician-diagnosed depression, which has suggested that this measure is valid for application in further cohort studies [34]. Finally, the subjective perception of job insecurity should be mentioned as a further limitation. However, research suggests that job insecurity as perceived by employees reflects the national economic situation [35,36]. Importantly, the results may be different in other social systems that may buffer or increase the health effects of unemployment [18].

These limitations are balanced by several strengths. The major strengths of this study are that it focused on mental health and related it to employment conditions using a large sample from Germany with national representativeness; therefore, our findings could be generalized to the whole country. Moreover, it was possible to control for a wide range of relevant confounding variables. In addition, we also conducted a sensitivity analysis, using different cut-points for the categorization of lifetime unemployment ((1) no unemployment experience, (2) short-term unemployment: <24 months, and (3) long-term unemployment experience: >24 months and job insecurity). The strongest health effect was observed for the category “long-term unemployment”, which was defined as cumulative unemployment time over 24 months (detailed data can be delivered on request). Finally, to our knowledge, this is the first study to test the separate and combined effects of job insecurity and lifetime unemployment experience in explaining the elevated risks for physician-diagnosed depression.

## 5. Conclusions

This longitudinal study indicated a strong effect of lifetime unemployment experience and job insecurity on the increased risk of depression. Even while employed, past long-term unemployment experience was related to a higher risk of depression, with the strongest association in those reporting job insecurity in their current position. The results emphasized the importance of employment and job security for mental health, as a target for health promotion and labor market social policy.

## Figures and Tables

**Table 1:** Characteristics of study sample at baseline (*n* = 7073).

Characteristics	Unemployment Experience		*p*	Job Insecurity		*p*
	Never or Short-Term (*n* = 6412)	Long-Term (*n* = 661)		None or Low (*n* = 5428)	High (*n* = 1645)	
Continuous variables (mean, SD)						
Age (years)	43.40, 11.11	45.35, 9.72	<0.0001	44.02, 10.89	42.15, 11.24	<0.0001
Years of education	13.00, 2.76	11.77, 2.28	<0.0001	13.06, 2.81	12.30, 2.43	<0.0001
Net income per month	1798.31, 1355.96	1020.72, 654.99	<0.0001	1831.01, 1377.36	1377.96, 1068.83	<0.0001
Categorical variables (*n*, %)						
Gender:						
Men	3390, 52.87%	291, 44.02%	<0.0001	2851, 52.52%	830, 50.46%	0.1413
Women	3022, 47.13%	370, 55.98%		2577, 47.48%	815, 49.54%	
Marital status:						
Unmarried	1585, 24.72%	141, 21.33%	<0.0001	1258, 23.18%	468, 28.45%	<0.0001
Separated/divorced/widowed	527, 8.22%	103, 15.58%		474, 8.73%	156, 9.48%	
Married	4300, 67.06%	417, 63.09%		3696, 68.09%	1021, 62.07%	
Smoking:						
No	4549, 70.95%	363, 54.92%	<0.0001	3832, 70.60%	1080, 65.65%	0.0001
Yes	1863, 29.05%	298, 45.08%		1596, 29.40%	565, 34.35%	
Alcohol consumption:						
Occasionally/seldom/never	5099, 79.52%	558, 84.42%	0.0027	4305, 79.31%	1352, 82.19%	0.0106
Regularly	1313, 20.48%	103, 15.58%		1123, 20.69%	293, 17.81%	
Sport:						
Never	1670, 26.04%	296, 44.78%	<0.0001	1458, 26.86%	508, 30.88%	<0.0001
Less than once a week	1979, 30.86%	204, 30.86%		1623, 29.90%	560, 34.04%	
Once a week or more	2763, 43.10%	161, 24.36%		2347, 43.24%	577, 35.08%	
BMI:						
Normal	3088, 48.16%	306, 46.29%	0.0042	2608, 48.05%	786, 47.78%	0.0921
Overweight	2346, 36.59%	222, 33.59%		1994, 36.74%	574, 34.89%	
Obese	978, 15.25%	133, 20.12%		826, 15.21%	285, 17.33%	
Contract:						
Permanent	4953, 77.25%	432, 65.36%	<0.0001	4243, 78.17%	1142, 69.42%	<0.0001
Non-permanent	1459, 22.75%	229, 34.64%		1185, 21.83%	503, 30.58%	
Self-rated health:						
Good	5819, 90.75%	556, 84.11%	<0.0001	4947, 91.14%	1428, 86.81%	<0.0001
Poor	593, 9.25%	105, 15.89%		481, 8.86%	217, 13.19%	

Differences were determined by Student’s *t* test or Chi-square test.

**Table 2:** Separate effects of unemployment experience and job insecurity at baseline on depression at follow-up (*n* = 7073).

		Incident Depression *n* (%)	Model I RR (95% CI)	Model II RR (95% CI)	Model III RR (95% CI)	Model IV RR (95% CI)
Unemployment experience in the past (years)	Never or short-term	203 (3.17%)	1.00	1.00	1.00	1.00
	Long-term	41 (6.20%)	1.82 (1.31, 2.54)	1.80 (1.28, 2.52)	1.70 (1.20, 2.39)	1.64 (1.16, 2.31)
Job insecurity in the future (0–100%)	None or low	166 (3.06%)	1.00	1.00	1.00	1.00
	High	78 (4.74%)	1.57 (1.20, 2.04)	1.54 (1.18, 2.02)	1.54 (1.18, 2.01)	1.48 (1.13, 1.92)

Model I: adjustment for age, and gender at baseline; Model II: additional adjustment for marital status, education, income, and contract type at baseline; Model III: additional adjustment for smoking, alcohol consumption, physical exercise, and BMI at baseline; Model IV: additional adjustment for self-rated health at baseline.

**Table 3:** Combined effects of unemployment experience and job insecurity at baseline on incident depression at follow-up (*n* = 7073).

Unemployment Experience in the Past	Job Insecurity in the Future	Incident Depression *n* (%)	Model I RR (95% CI)	Model II RR (95% CI)	Model III RR (95% CI)	Model IV RR (95% CI)
Never or short-term	None or low	143 (2.85%)	1.00	1.00	1.00	1.00
Never or short-term	High	60 (4.28%)	1.52 (1.13, 2.04)	1.54 (1.14, 2.08)	1.53 (1.14, 2.07)	1.48 (1.10, 1.99)
Long-term	None or low	23 (5.50%)	1.79 (1.16, 2.76)	1.84 (1.19, 2.85)	1.72 (1.11, 2.67)	1.67 (1.07, 2.60)
Long-term	High	18 (7.41%)	2.44 (1.52, 3.92)	2.38 (1.45, 3.91)	2.30 (1.40, 3.79)	2.15 (1.32, 3.52)

Model I: adjustment for age, and gender at baseline; Model II: additional adjustment for marital status, education, income, and contract type at baseline; Model III: additional adjustment for smoking, alcohol consumption, physical exercise, and BMI at baseline; Model IV: additional adjustment for self-rated health at baseline.
